# Fruit fly identification, population dynamics and fruit damage during fruiting seasons of sweet oranges in Rusitu Valley, Zimbabwe

**DOI:** 10.1038/s41598-019-50001-w

**Published:** 2019-09-19

**Authors:** Stephen T. Musasa, Arnold B. Mashingaidze, Robert Musundire, Ana A. R. M. Aguiar, Jorge Vieira, Cristina P. Vieira

**Affiliations:** 1 Chinhoyi University of Technology, School of Agricultural Sciences and Technology, Department of Crop Science and Postharvest Technology, Chinhoyi, Zimbabwe; 2 Universidade do Porto, LAQV, REQUIMTE, GreenUPorto-UP & DGAOT, Faculdade de Ciências, Campus Agrário de Vairão, Vila do Conde, Portugal; 3 Instituto de Biologia Molecular e Celular (IBMC), Universidade do Porto, Rua Alfredo Allen, 208, 4200-135 Porto, Portugal; 4 Instituto de Investigação e Inovação em Saúde (I3S), Universidade do Porto, Rua Alfredo Allen, 208, 4200-135 Porto, Portugal

**Keywords:** Invasive species, Invasive species

## Abstract

In 2003, the pest species *Bactrocera dorsalis* (Hendel) was reported for the first time in Kenya, Africa, and subsequently on many other African countries. In this work, 20 locations along the Rusitu Valley (Zimbabwe) were sampled in 2014 during the sweet oranges fruiting seasons, to verify the fruit fly taxonomy, invasion source, population dynamics, and fruit damage. The trapped fruit flies were identified using morphological traits and molecular techniques, as *B. dorsalis*. The haplotype network analysis revealed that Zimbabwe *COI* sequences were identical to other African *B. dorsalis* sequences. Fruit fly trappings per day varied during the year, although it remained always high. The same applies to fruit damage, most likely due to the permanent availability of cultivated and wild fruit varieties during the year. Rusitu Valley was invaded by *B. dorsalis*, most likely from neighbouring countries. Ten years after the first report in Kenya, the complete or near complete invasion of Africa has been achieved by *B. dorsalis*. In northern Africa the distribution is clearly limited by the Sahara desert. The large population size, the polyphagous nature of the species, and the continuous availability of suitable host fruit species during the year complicates the eradication of this species.

## Introduction

Fruit and vegetable production is one of the fast-growing agricultural sectors in Zimbabwe, providing income to farmers. One of the major fruit crops which strengthen the livelihood of many farmers in Zimbabwe is sweet orange (*Citrus sinensis*). Oranges are mainly grown in areas within or surrounding Rusitu, Limpopo, Save, and Mazowe Valleys in Zimbabwe. The production of oranges in Zimbabwe is mainly for consumption as fresh fruit and/or juice in domestic and export markets. Orange production in Zimbabwe has been increasing since 1980, reaching a peak of 116078 tonnes in 2003^1^. Nevertheless, orange production declined to 97512 tonnes in 2011^1^, mainly because of challenges related to poor pre-and post harvest management^2^. The decline was further exacerbated by the recent invasion of Zimbabwe by alien fruit flies (Diptera: Tephritidae), known as African invader fruit flies (*B. dorsalis* (Hendel)) in 2010^3^. Although the presence of *B. dorsalis* (Hendel) in Zimbabwe was first recorded in 2010^3^, it was only recognized in 2012 by the government’s Plant Quarantine Services Institute [Chikwenhere, G., personal communication]. The *B. dorsalis* species was first reported in Kenya in 2003^3,4^, but, it has already become a pest of major concern to fruit growers in many parts of Africa.

The African invader fruit fly species belongs to *B. dorsalis* complex and is devastating to crops causing millions in lost production each year^5–9^. The *Bactrocera* genus harbours over 75 species with broad but, essentially allopatric distributions with regions of transition occurring around the south east of Asia^10–14^. Studies have revealed that amongst these species, the most damaging one is *B. dorsalis*^15–17^ and there are some morphological differences in their populations^7–10^.

Since its arrival in Africa, *B. dorsalis* is believed to have rapidly expanded its range at an alarming rate. It is now reported to have spread throughout the equatorial, tropical and subtropical regions of Africa but, not yet reported in Malawi, Somalia, and Lesotho^3^. This rapid expansion is likely due to its polyphagous nature and high reproductive potential. Indeed, *B. dorsalis* is known to attack at least 46 host plants, including many commercially grown fruit crops such as mango, oranges, guava, cucurbit, papaya, and avocado, as well as many other species indigenous to Africa^15,17–19^. In some African regions, such as Tanzania, Kenya, Benin, and Cameroon, *B. dorsalis* is now the major pest on host species such as mangoes, having displaced the native *Ceratitis* species as the main pest^16,17,20^. Reports indicate that horticultural yield losses averaging 15–50% are caused by *B. dorsalis* in several African countries^21^. The damaging activity of *B. dorsalis* is mainly due to female oviposition since they use their ovipositor to lay eggs in clutches under the skin of the fruit^22^. By this process, spoilage microbes are introduced into the fruit causing the fruit to breakdown and rot. Once in the fruit, the eggs hatch into larvae or maggots. It is the decaying flesh that provides food for the larvae or maggots. When fully grown, the larvae escape from the fruit, burrow into the soil or organic matter and transform into pupae. Twenty days after eggs are laid the adult fruit fly emerges from the puparium^22,23^, and the cycle restarts.

The details of the African invasion are currently being worked out but, so far, two clear main outbreaks have been recognized, with an east African origin, likely in Kenya and Tanzania^24^. There is an evident absence of geographical structure across Africa, with the exception of Nigeria that could represent a third independent outbreak^24^. It should be noted that, according to other studies^13,19^, there are likely suitable habitats for *B. dorsalis* in other regions of the world such as the neo-tropics, Europe and Australia, and outbreaks have been reported in California, USA, in 2006 and 2012^25^. Understanding how Africa has been invaded and how this species has become established in a large geographic region may help in the formulation of strategies to eradicate this species from Africa and help prevent future invasions of other geographic regions.

Evidence for introgression between *B. dorsalis* and *B. kandiensis* has been previously reported^8^. Introgression between the Australian species *B. tryoni* and *B. neohumeralis* has been proposed as a potential adaptive mechanism allowing the expansion of *B. tryoni* into new climatic regions^26^, and thus the same could be true for *B. dorsalis*. *B. kandiensis* is recorded, so far, from Sri Lanka only, but, it may occur sympatrically with *B. dorsalis* in India and Myanmar^8^. The source of the two clear east African outbreaks is very likely Asian countries east of Sri Lanka, India and Myanmar, since only a very small percentage of African flies, show evidence of introgression. Verification of the taxonomy, population dynamics, fruit damage, and invasion source of *B. dorsalis* populations that have invaded Zimbabwean territories is critical for development of management, control and/or eradication measures. Understanding the fruit fly population dynamics and invasion pathway will also assist in drawing the population structure of *B. dorsalis* complex. In turn, this will help in the implementation of global policies which reduce the expansion of this species. Therefore, in this work we identified the fruit fly species in Zimbabwe’s Rusitu Valley, determined possible sources of the invasion, and established the population dynamics and fruit damage in this region.

## Results and Discussion

### Fruit fly species identification

**Figure 1:**
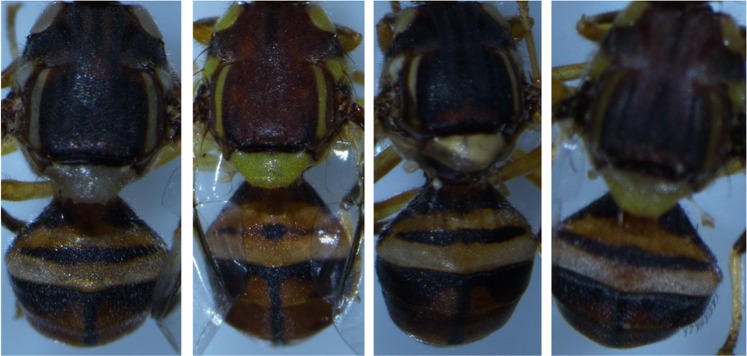
Morphological features of *B. dorsalis* adult males. The four different individuals show variation in body colour and stripes.

The male fruit fly specimens from Rusitu Valley possessed a very narrow coastal band and anal streak, scutum which is dark orange-brown with a black lanceolate pattern, narrow lateral post-sutural vittae and abdominal tergites III–V with a dark ‘T’ pattern and narrow dark lateral markings on all three terga, as shown in Fig. 1. These morphological features are congruent with the identification key for *B. dorsalis*^4^.

The right-hand wings of the trapped fruit flies were also analysed and found to be compatible with the 15 wing landmarks identification key for *B. dorsalis*^7^ (Table 1; Supplementary Fig. S1). A mean wing length of 4.3 mm (±0.2) was observed for Zimbabwean populations of *B. dorsalis*, slightly lower than 6 mm and 7.3 mm observed, respectively for *B. dorsalis* of Asian and American origin^27,28^. This also supports the idea of morphological differences across *B. dorsalis* complex as recently observed by several authors^5,7–9,29^. It should be noted that about 16% of the sampled individuals have a broken dorsal stripe thus, resembling *B. kandiensis*.

**Table 1:** The 15 wing landmarks for *B. dorsalis*.

Landmark Postition	Description
1	Basal junction of veins of cell
2	Anterior-most point of the suture located towards the base of vein
3	Inner antero-distal corner of cell
4	Junction of veins A_1_ and CuA_2_
5	Junction of CuA_1_ and CuA_2_
6	Junction of vein CuA_1_ and dm-bm cross vein
7	Junction of vein M and dm-bm cross-vein
8	Junction of vein CuA_1_ and dm-cu
9	Junction of vein M and dm-cu
10	Junction of vein M and r-m cross-vein
11	junction of vein R_4+5_ and r-m cross-vein
12	Junction of vein R_1_ and costal vein
13	Termination of vein M
14	Termination of vein R_4+5_
15	Termination of vein R_2+3_

### Confirmation of fruit fly identification and invasion pathway

**Figure 2:**
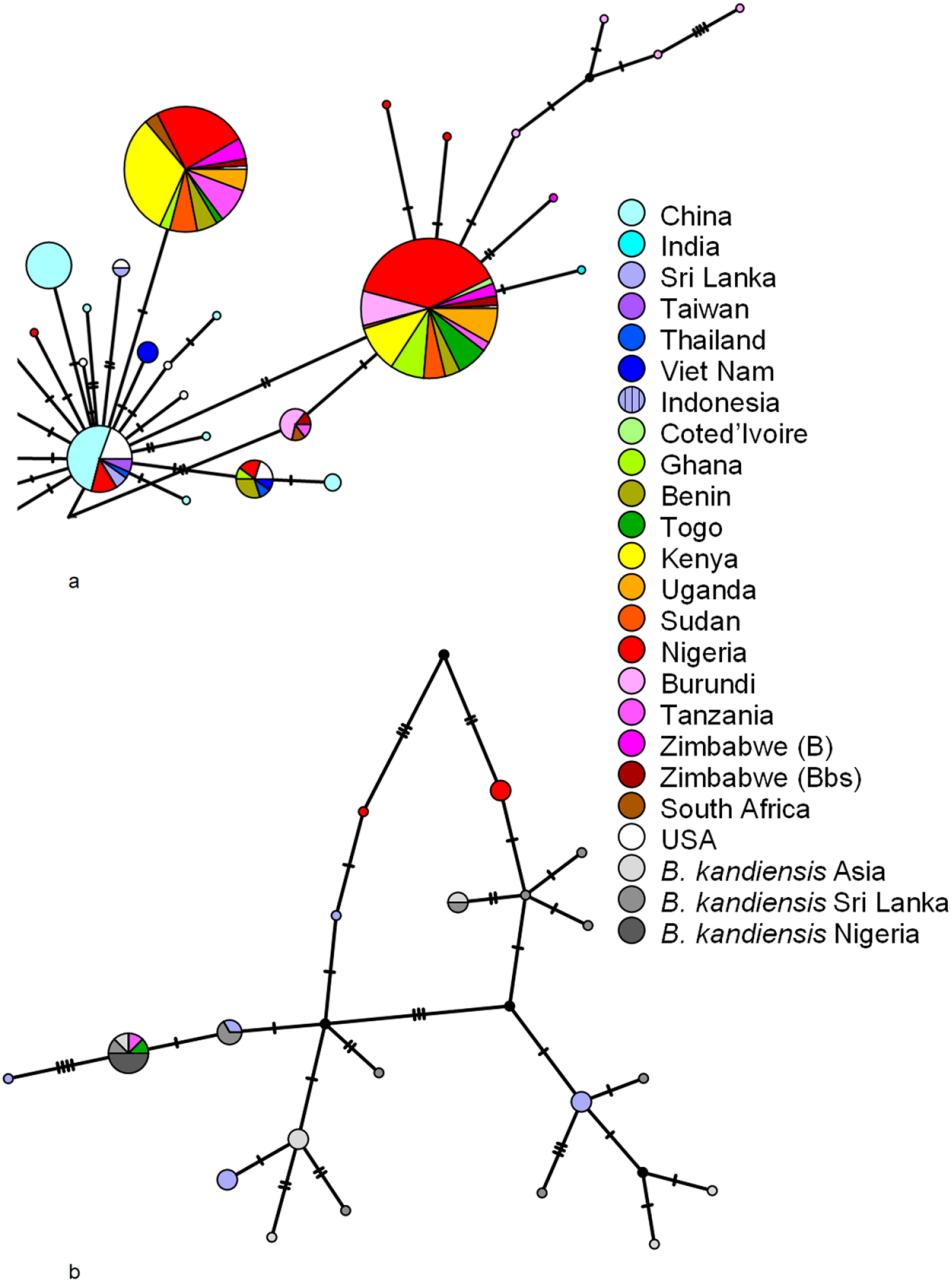
Partial view of the TCS haplotype network of 533 *Bactrocera* sequences. (**a**) The Zimbabwean sample (B–random sample; Bbs–sample resembling *B. kandiensis*). (**b**) The *B. kandiensis* sample. The full image is available as Supplementary Fig. S2.

In order to have a molecular confirmation of the species identification and infer the source of Zimbabwe population as an independent invasion or from neighbouring countries, the mtDNA *COI* region was amplified, cloned and sequenced from 11 randomly chosen individuals (assigned as B in Fig. 2a). Additional sequences were retrieved from GenBank to perform a Haplotype Network analysis (since mtDNA is non-recombining) with 533 sequences for which the origin of the sample is declared in GenBank nucleotide records (the alignment is 658 bp long; it also includes a non-random sample of six individuals from Zimbabwe (assigned as Bbs in Fig. 2a; Supplementary Fig. S2). All 11 sequences from Zimbabwe flies cluster into two large groups, namely, six sequences are identical to 101 *B. dorsalis* sequences available in GenBank, four are identical to 129 *B. dorsalis* sequences available in GenBank and one sequence shows three nucleotide differences from the latter group. Therefore, the collected flies are clearly *B. dorsalis*. Moreover, all Zimbabwean *B. dorsalis* samples cluster into the two clades that likely represent the two original main outbreaks with an east African origin, probably from Kenya and Tanzania^24^. Therefore, the source of the Zimbabwean invasion is likely from neighbouring countries and not from an independent invasion. Kenya and Tanzania are the most probable source of Zimbabwean invasion since several fruit fly outbreaks were recorded earlier in these two countries. In addition, no control and/or management programs were implemented in these countries to combat the African invader fruit flies^3,15^. It should be noted that there is an evident absence of geographical structure in Africa, with the exception of Nigeria that could represent a third independent outbreak^24^. Our analyses support this hypothesis since 10 out of 11 *B. dorsalis* sequences from Zimbabwe are identical to *B. dorsalis* sequences already reported from other African regions. As expected, given that the two original invasions likely involved a small number of individuals, for the Zimbabwe population, polymorphism levels are low (π = 0.0038; Θ = 0.0031; 11 sequences analysed presenting 6 segregating sites). Similar values have been reported using other molecular markers for African populations^30^. Invasive populations in Africa and also in Hawaii are those presenting lower genetic diversity. In our sample Tajima’s D is, however, non-significant (D = 0.96038; P > 0.05) showing that there is no excess of rare polymorphisms as expected under a scenario of population growth. Similar results are reported for another African sample for different loci^30^. The low polymorphism level implies that most sequences are identical to those already obtained or differ by a few mutations, thus, a very large sample would not be more informative.

In our haplotype network analysis, Nigerian *B. dorsalis* samples cluster with *B. kandiensis* samples and with *B. dorsalis* samples from Sri Lanka, India, and Myanmar (Fig. 2b; Supplementary Fig. S2). This observation is compatible with the evidence for introgression that has been reported between *B. dorsalis* and *B. kandiensis*^8^, but also shows that the Nigerian population is the result of an independent invasion from Sri Lanka, India or Myanmar. This could also explain why there is an evident absence of geographical structure across Africa, with the exception of Nigeria^24^. The limited distribution of the individuals showing evidence for introgression suggests that in contrast to what happens with *B. tryoni* in Australia^26^, it is unlikely that these individuals have any advantage that would allow their expansion.

While performing the phenotypic characterization, we noted that about 16% of the sampled individuals have a broken dorsal stripe thus resembling *B. kandiensis*. Since these individuals are not present at a very high frequency, it was possible that such individuals were not represented in the random sample of 11 individuals that we analyzed molecularly. Therefore, we also characterized six individuals with a broken dorsal stripe using molecular methods. Nevertheless, the network analysis that we performed clearly shows that this biased sample is not very different from the random sample that was originally characterized (Fig. 2a; Supplementary Fig. S2). Therefore, there is no evidence for introgression in the Zimbabwean sample.

**Figure 3:**
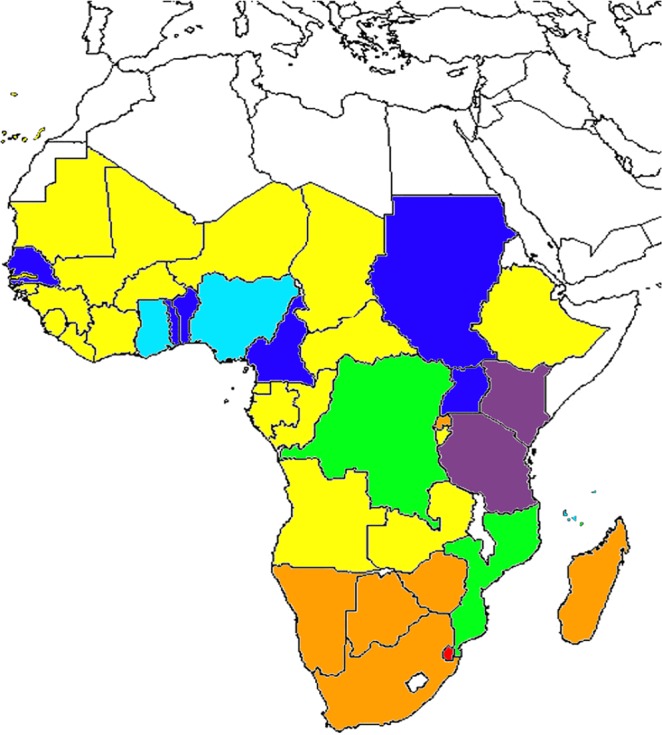
History of the *Bactrocera dorsalis* African invasion. Purple–2003; Dark blue–2004; Light blue–2005; Green–2007; Yellow–2008; Orange–2010; Red–2013.

The positive morphological and molecular identification of Zimbabwe fruit flies as *B. dorsalis* allows us to join one more piece into the puzzle of the African invasion by this species. Figure 3 shows where and when, in Africa, *B. dorsalis* has been reported. With the exception of Lesotho, Malawi, and Somalia, *B. dorsalis* has now been reported in every African country where suitable habitat has been predicted for this species^19^. It should be noted that it is unclear whether in Lesotho, Malawi, and Somalia there was an attempt to look for *B. dorsalis*. Therefore, 10 years have been enough for the complete or near complete invasion of Africa by this species, since in northern Africa the distribution is clearly limited by the Sahara desert. In some regions, such as Tanzania, *B. dorsalis* is now the major pest species in hosts such as mangoes, having displaced the native *Ceratitis* species as the main pest^16,17^. Since *B. dorsalis* African invasion appears to be the result of a single recent invasion, with little or no subsequent gene flow with source populations in South Asia^9,24,30^, it is unexpected to see marked morphological and biological differences between these geographic populations.

### Fruit fly population dynamics and fruit damage

**Figure 4:**
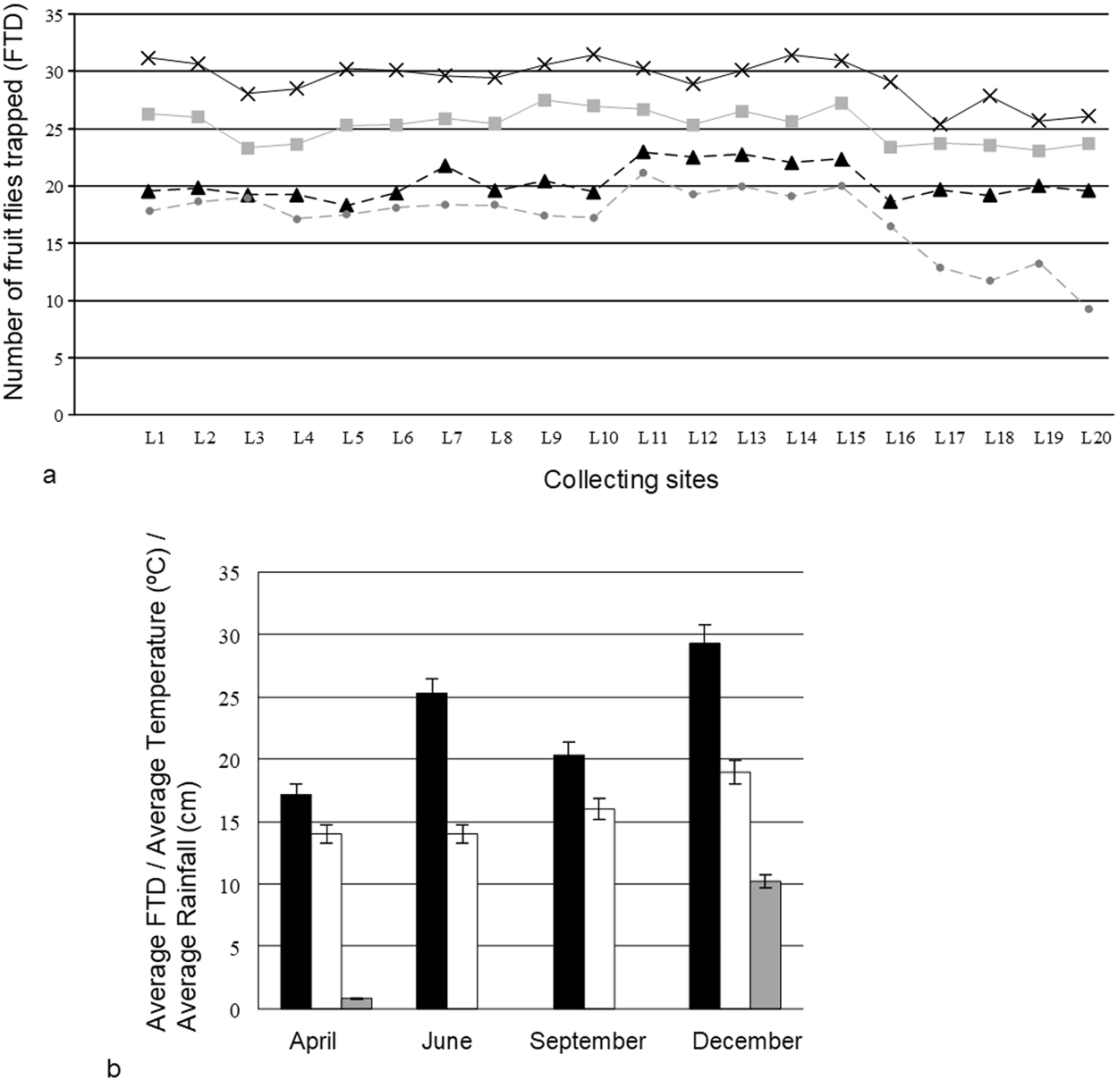
Fruit flies per trap per day (FTD) and weather data. (**a**) FTD data for all locations (assigned as L1 to L20) for the months of April (black line with x), June (grey line with squares), September (black dotted line with triangles), and December (grey dotted line with dots). (**b**)Monthly averages of FTD (in black), mean temperature (°C; in white), mean rainfall (cm; in grey) for April, June, September, and December.

Zimbabwean farmers from the Rusitu Valley also perceive that fruit flies are causing havoc in post harvest management of their fruits. The livelihood of most rural district population in Rusitu Valley depends on fruit crop production. One of the major fruit crops which strengthen their livelihood is sweet orange. Reports indicate that farmers are facing orange postharvest losses estimated to be more than 30% and most of these losses are being caused by fruit flies^2^. Therefore, in this work, we also looked at the population dynamics of *B. dorsalis* and fruit damage during the time of sweet orange ripening. There are differences in the number of fruit flies trapped per day (FTD) during the four months in which farmers harvest sweet oranges (Fig. 4a). December had the highest FTD in all the trap positions (Fig. 4b) and this could be justified by prevailing weather conditions of relatively high humidity and temperature. Indeed, in 2014, April and June recorded a mean temperature of 14 °C, September 16 °C, and December was humid with a mean temperature of 19 °C (Fig. 4b). A statistically significant positive correlation (r = 0.685; P < 0.05) existed between FTD and temperature. The weather conditions prevailing in December coupled with the fruiting of mango fruits can be linked to higher FTD. Previous studies by other researchers supported evidence that fluctuations in temperature in horticultural agro-ecosystems help to explain shifts in dominance of *B. dorsalis* in many parts of Africa^31^. The locations 16, 17, 18, 19, and 20 positioned to the South-western side of the valley recorded low FTD values compared to other trapping locations and this part of the valley is not always evergreen compared to the North-eastern sides (Fig. 4a). In this part of the valley, fruit production is generally low and farmers grow tea and coffee as their main source of livelihood^32,33^. It is also important to note that the month of April recorded very low FTD values, lower than June, September, and December (Fig. 4b). This could be linked to decreasing temperature and humidity since previous studies have shown that fruit flies change behaviour in preparation for late summer^34,35^. Another factor that could have contributed to low FTDs in April is a shift in the availability of quality food for the fruit flies. Although *B. dorsalis* is a polyphagous species, they do prefer mango fruits more than oranges^17,20^. In April, in Rusitu Valley, only local varieties of oranges will be ripening unlike in other studied months. Spatial variation existed in FTD (Kruskal-Wallis Test; P < 0.05, DF = 3), as well as temporal variation (Friedman ANOVA; F = 573.812, P < 0.05, DF = 3). Likewise, the Mann-Whitney and Wilcoxon pair wise comparison tests confirmed significant subtle spatio-temporal differences (P < 0.05) in FTD for April versus June, April versus September, and April versus December. However, there were no pair wise statistical differences in FTD for December versus June, and December versus September.

**Figure 5:**
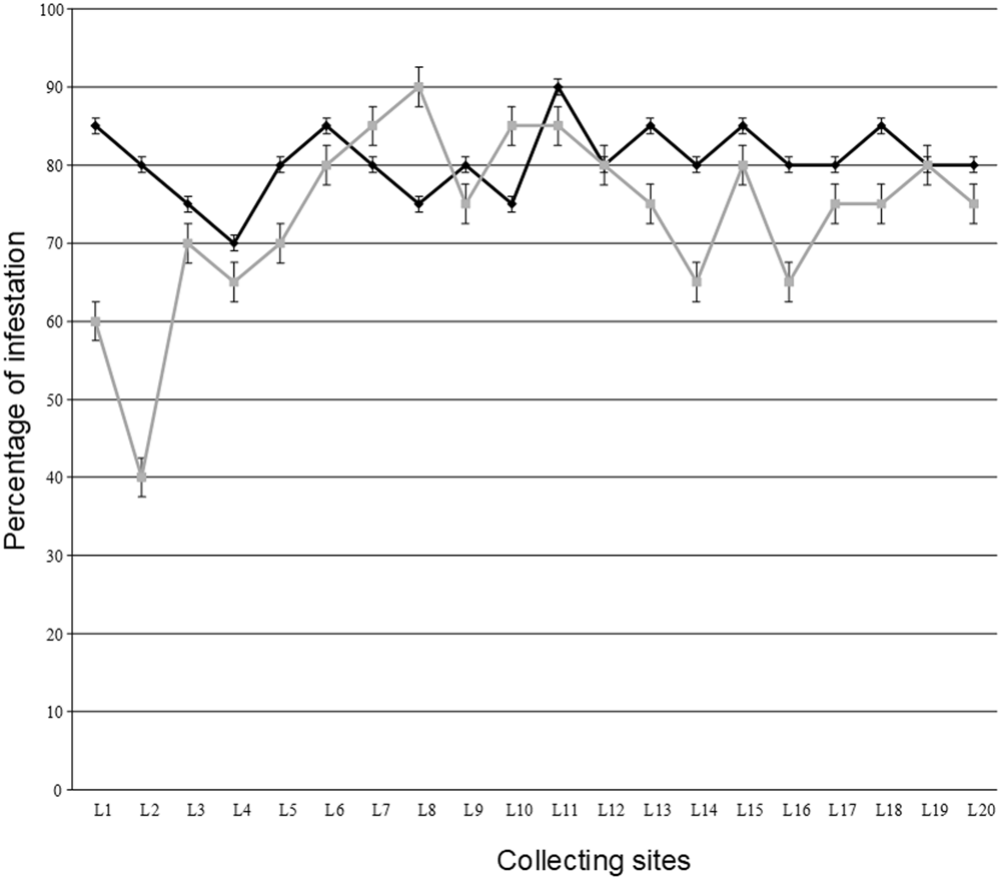
Fruit fly infestation per locality. Percentage of fruits showing oviposition marks for June (black line with diamonds) and December (grey line with squares) 2014.

When looking to fly infestation, a picture similar to that obtained for the FTD analysis is obtained (Fig. 5). The means for infestations were 80.50% (N = 20, standard error of 1.02) and 73.75% (N = 20, standard error of 2.51) for June and December respectively. Therefore, although December had a higher density of fruit flies than June, the percentage of fruits showing ovipositor marks was higher in June than in December (Mann-Whitney test; P < 0.05; Fig. 5). The high infestation rates imply a loss in orange production since most fruit damage is caused during oviposition by the introduction of spoilage microbes into the fruit. The ovipositor marks also reduce the aesthetic value of oranges at markets.

**Figure 6:**
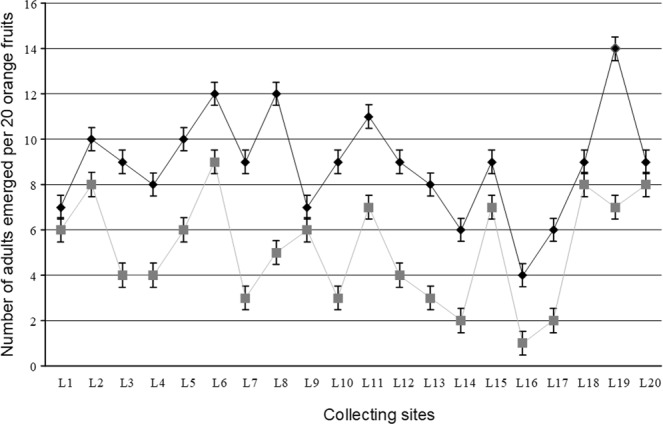
Emerged adult fruit flies per location. Average number of adults emerged per 20 fruit for the months of June (black line with diamonds) and December (grey line with squares) 2014.

Despite the high infestation rates, a low number of adults emerged per fruit were observed (Fig. 6). The mean number of emerged adults/fruit was 0.445 ± 0.118 and 0.258 ± 0.116 adults/fruit, respectively for the months of June and December. These values are significantly different (T-test; P < 0.0001 at *α* = 0.05, *t* = 4.955 with 38 degrees of freedom). The assumption that the data were sampled from populations that follow Gaussian distribution was tested using the Kolmogorov and Smirnov (KS) method (P > 0.10) for both June and December. The results support the idea that sometimes fruit flies make pseudo-punctures (punctures without eggs) which increases post harvest losses through a reduction in the market value of fruits^36^. These results also support the suggestion that oranges are possible hosts for *B. dorsalis* though they are not the most preferred^17^. More studies are needed to determine the most preferred hosts of *B. dorsalis* in the valley.

## Conclusions

In conclusion, Rusitu Valley has been colonised by *B. dorsalis*, with high numbers of individuals observed during the year that cause a high degree of damage in fruits. The high numbers of individuals are likely maintained by the permanent availability of cultivated and wild fruit varieties along the year. In light of study findings, the invasion of Zimbabwe’s Rusitu Valley was not an independent invasion. The source of the invasion in Zimbabwe is likely from neighbouring countries. Zimbabwe’s invasion pathway is linked to the two reported clear east African outbreaks whose source was revealed to be Asian countries east of Sri Lanka, India, and Myanmar. It can be noted that, ten years after the first report in Kenya of *B. dorsalis*, the complete or near complete invasion of Africa has been achieved since in northern Africa the distribution is clearly limited by the Sahara desert. The large population size, the polyphagous nature of the species, and the availability of suitable fruits along the year make it difficult to fight this species.

## Materials and Methods

### Description of the study area

Zimbabwe’s Rusitu Valley (20°S 032°E), on an altitude of about 460 m above sea level, receives moderately high rainfall (>1000 mm) and temperatures (>19 °C) almost throughout the year, thus the valley is characterised by a warm and humid climate^32,37^. The soil particles are well graded and consolidated making them less vulnerable to erosion, enabling farmers to plough and grow fruit crops on slopes and hilly places^38^. These conditions make Rusitu Valley suitable for the production of a wide variety of fruits which include bananas, oranges, mandarins, and mangoes.

### Study approach

The study addressed three important issues in Rusitu Valley’s sweet orange production chain. These are: (1) identification of fruit fly species, (2) determination of possible sources of the invasion, and (3) establishing the population dynamics and fruit damage in Rusitu Valley, Zimbabwe. Permission for field studies in Rusitu Valley was sought from communal traditional leaders.

### Data sources

**Figure 7:**
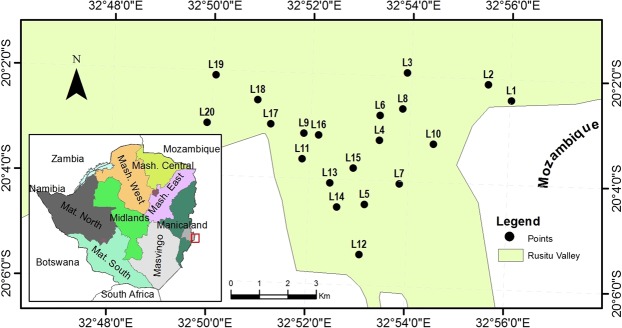
Map of Zimbabwe, showing the sampling block and geographical location of collection sites, coded L1 to L20. The map was constructed using datum GCS-Arc-1950WKID:4209.

The fruit fly infestation, emergence, and trapping experiments were all carried out from the four sweet orange producing wards in Rusitu Valley to constitute a sampling block (Fig. 7). From the sampling block, twenty locations were selected using snowball sampling technique because of the steep and hilly terrain in the Rusitu Valley that made access to a random sample extremely difficult^39^. These locations were backyard orchards less than half of an acre (>2023.5 m^2^) and each location had on average 25 trees. At these locations, trapping measurements were done once per month for four months. Infestation and emergence measurements were done once per month for two months. The assumption was that weather conditions were uniform across the sampling block and that they were varying according to month of the year. Weather data used in the study were drawn from Chisengu Weather Station records available online; https://www.wunderground.com/zw/chisengu.

### Trapping of *B. dorsalis* male fruit flies

Unlike banana cultivars which are available throughout the year, orange fruiting seasons in Rusitu Valley of Zimbabwe is in April, June, September, and December depending on the orange cultivars. Thus in this study, trapping was performed in 2014, on orange trees in these four months. Mango fruits are only available from December up to early February also depending on the cultivars^40^, and thus flies collected in April, June and September are likely to represent a good sample of fruit flies infesting mainly oranges. Nevertheless, *B. dorsalis* species are highly polyphagous, and thus we cannot exclude the possibility that these flies are completing their life cycle on other Zimbabwean native plant species as well. In order to trap flies, methyl-eugenol (here after ME) which is a known para-pheromone that has a powerful attraction to the adult male fruit flies of the *B. dorsalis* complex was used. Twenty traps were prepared using locally available empty 2 litre polyethylene (PET) bottle containers. Each bottle was cleaned thoroughly with water and cut at the circular/curved position which signified the starting point of the cone so that the cone is inverted and placed into the container with the lid opening pointing towards the base of the container. The flashing cut edges were sealed using a strong adhesive cellulose tape. Two holes (~3 cm × 2 cm) were cut on the sides of the container at an orientation directly opposite to each other. A solution of ME and Malathion 50% EC was prepared (5 ml ME + 5 ml Malathion 50% EC + 990 ml water) and a cotton swab dipped into the solution for one minute. The cotton swab was then placed into the container to constitute a trap, which was then hung onto the canopy of the host orange fruit tree (Supplementary Fig. S3). One trap was placed per study location. Trapped flies were collected and counted after 30 days of setting the trap. The counted flies were pooled and kept in a dry state at room temperature in the Post harvest Laboratory, Department of Crop Science and Postharvest Technology at Chinhoyi University of Technology, Zimbabwe for use in morphological and molecular identification experiments.

### Morphological description of fruit flies

A total of 100 fruit fly specimens were randomly selected from the pooled population of fruit flies. The specimens were examined using stereomicroscope (Nikon SMZ1000) for the three major *B. dorsalis* discriminating morphological features which are: clear wings with a continuous dark coastal band, two yellow stripes on the thorax, and a dark T-shaped marking on abdomen^4,7^. The right-hand wings of the 100 specimens were also analysed for wing length^40^ and 15 wing landmarks identification key^7^ (Supplementary Fig. S1), using Nikon NIS-Elements version 3.2 (Nikon Instruments, Japan).

### Confirmation of fruit fly identification and invasion sources

#### DNA extraction and PCR amplification

A sample of 11 individual fruit flies was randomly selected from the pooled population^41^. Six individuals having a broken dorsal stripe, thus resembling *B. kandiensis*, were also analysed, to address introgression in Zimbabwe. The genomic DNA of each selected individual was extracted using the QIAamp DNA Mini Kit (250) as per manufacturer’s instructions (Quiagen, Germany). The genomic DNA was stored at −20 °C.

The PCR experiments were carried out using the universal primers developed by Folmer *et al*.^42^; primers LCO (5′-1490GGTCAACAAATCATAAAGATATTGG-3′) and HCO (5′-2198TAAACTTCAGGGTGACCAAAAAATCA-3′) to amplify an approximately 700 base pair fragments of the *COI* gene. The reaction mixture of 20 µl for each sample consisted of 9.2 µl H_2_O, 2 µl Buffer (10X), 2 µl of dNTP mix (100 mM), 1 µl of Forward primer (100 mM), 1 µl of Reverse primer (100 mM), 2.4 µl MgCl_2_ (25 mM), 0.4 µl Taq DNA polymerase (5 U/µl) and 1 µg of genomic DNA. Standard cycling conditions were 96 °C for 2 minutes for initial denaturation of DNA, followed by 40 cycles of denaturation at 95 °C for 30 seconds; 50 °C for 45 seconds and 72 °C for 2 minutes respectively, and strand extension at 72 °C for 5 minutes. PCR products were separated by electrophoresis on a 1.5% agarose gel in 1 × TBE buffer (pH 8.0) and the DNA amplification products were visualised using GreenSafe Premium (Nzytech) staining, and a transilluminator. DNA was extracted from the gel using the QIAEX II Gel Extraction kit as per manufacturer’s instructions (Quiagen, Germany), and kept at −20 °C for cloning. The TOPO TA Cloning Kit for Sequencing (Invitrogen, California, USA) was used to clone the DNA fragments according to the manufacturer’s instructions. Plasmid DNA of three colonies was extracted using NZY Mini prep kit according to the manufacturer’s instructions (NZY tech, Portugal), and sequenced at StabVida (Lisbon) using the M13F and M13R universal primers, and the ABI Big Dye v1.1 chemistry (Applied Biosystems Europe, Spain) chemistry, as recommended by the manufacturers. Chromatograms were analysed using PROcessor of SEQuences version 2.91^43^, and for each individual, a consensus sequence was obtained in order to correct for possible errors that may occur during the PCR amplification step. *COI* gene sequences were submitted to GenBank.

#### Sequence analysis and inference of invasion sources

The 17 Zimbabwe sequences and 533 GenBank sequences from all over the world were aligned with Clustal Omega^44^. The haplotype network technique was used to infer the invasion sources of fruit flies in Zimbabwe. The haplotype network was obtained using the TCS method as implemented in PopArt (http://popart.otago.ac.nz)^45^. Summary statistics were calculated using DNAsp^31^.

### Fruit fly population dynamics and fruit damage

#### Fruit fly per trap per day

The number of flies per trap per day (hereafter FTD) was calculated using the following formula^46^:

$${\rm{FTD}}=\frac{{\rm{Total}}\,{\rm{number}}\,{\rm{of}}\,{\rm{flies}}\,{\rm{trapped}}}{{\rm{Number}}\,{\rm{of}}\,{\rm{trapping}}\,{\rm{days}}\times {\rm{Number}}\,{\rm{of}}\,{\rm{traps}}}$$

#### Infestation experiments

The infestation experiments were carried out in all the 20 locations during the months of June and December. A total of 20 mature oranges were randomly selected from orange trees at each study location avoiding old fallen fruits to reduce the possibility of picking fruit damaged by other ground-dwelling insects. Therefore, a total of 400 fruits were used for infestation experiments and they were not discarded but, kept for adult emergence experiments. Each selected fruit was visually examined with the aid of a hand lens to detect fresh oviposition marks that are not visible to the naked eye. The number of fruits showing oviposition marks was determined and the percentage infestation was calculated using the following formula^46^:

$${\rm{Infestation}}=\frac{{\rm{Number}}\,{\rm{of}}\,{\rm{fruits}}\,{\rm{showing}}\,{\rm{ovipositor}}\,{\rm{marks}}\times 100}{{\rm{Total}}\,{\rm{number}}\,{\rm{of}}\,{\rm{fruits}}\,{\rm{examined}}}$$

#### Adult fly emergence experiments

Sweet orange fruits were weighed to determine the average unit weight before being placed in rearing boxes. For each location, 20 fruits were used and to minimise difficulties in managing the experiments, four fruits were placed in one rearing box prepared using transparent plastic cube box containers and measuring 20 × 10 × 10 cm in length, width, and height respectively. Five rearing boxes were allocated per location. Each rearing container was lined with approximately 8 cm^3^ of insecticide-free dry sieved sand. Four orange fruits (from the same location) were placed in a rearing container and 1 mm mesh-size rayon cloth was used to tightly cover the container. Elastic rubber bands were used to tighten the rayon cover to prevent flies from entering or escaping the rearing container. These containers were placed in an airy room protected from ants for a period of four weeks and monitored on weekly intervals. At the end of the fourth week, the rearing boxes were carefully examined and all pupae and/or flies were collected. The average number of adult fruit flies that emerged per orange fruit per location was calculated as:

$${\rm{Flies}}\,{\rm{per}}\,{\rm{fruit}}\,{\rm{per}}\,{\rm{location}}=\frac{1}{5}\mathop{\sum }\limits_{i=1}^{5}\,(\frac{{\rm{collected}}\,{\rm{flies}}\,{\rm{in}}\,{\rm{box}}i}{4})$$

#### Statistical analysis

Stata11^47^ was used to determine spatial and temporal heterogeneity (variation) in FTD using non-parametric tests (Kruskal-Wallis and Friedman ANOVA) at 5% level of significance. GraphPad In Stat version 3.10 was used to analyse for normality, correlation and descriptive statistics on FTD, adult emergence, infestation, and weather data^48^.

## Supplementary information

Fruit fly identification, population dynamics and fruit damage during fruiting seasons of sweet oranges in Rusitu Valley, Zimbabwe

## Data Availability

The data sets used and/or analysed during the current study are available from the corresponding author on reasonable request.
