# Supplementary material for: Fruit fly identification, population dynamics and fruit damage during fruiting seasons of sweet oranges in Rusitu Valley, Zimbabwe
Source: Sci Rep. 2019 Sep 19;9:13578. doi: 10.1038/s41598-019-50001-w (PMC6753090; doi:10.1038/s41598-019-50001-w)
Supplement: Supplementary file 1 — Fruit fly identification, population dynamics and fruit damage during fruiting seasons of sweet oranges in Rusitu Valley, Zimbabwe [file 41598_2019_50001_MOESM1_ESM.pdf]

Fruit fly identification, population dynamics and fruit damage during fruiting seasons of sweet oranges in Rusitu Valley, Zimbabwe

Stephen T. Musasa, Arnold B. Mashingaidze, Robert Musundire, Ana A.R.M. Aguiar, Jorge Vieira, and Cristina P. Vieira

Supplementary Information

Supplementary Fig. S1. Right-hand wing of *B. dorsalis* showing the fifteen landmarks

Supplementary Fig. S2. TCS haplotype network of 533 *Bactrocera COI* sequences

Supplementary Fig. S3. Two litre polyethylene (PET) bottle trap used in Rusitu Valley to trap male adults of *B. dorsalis*

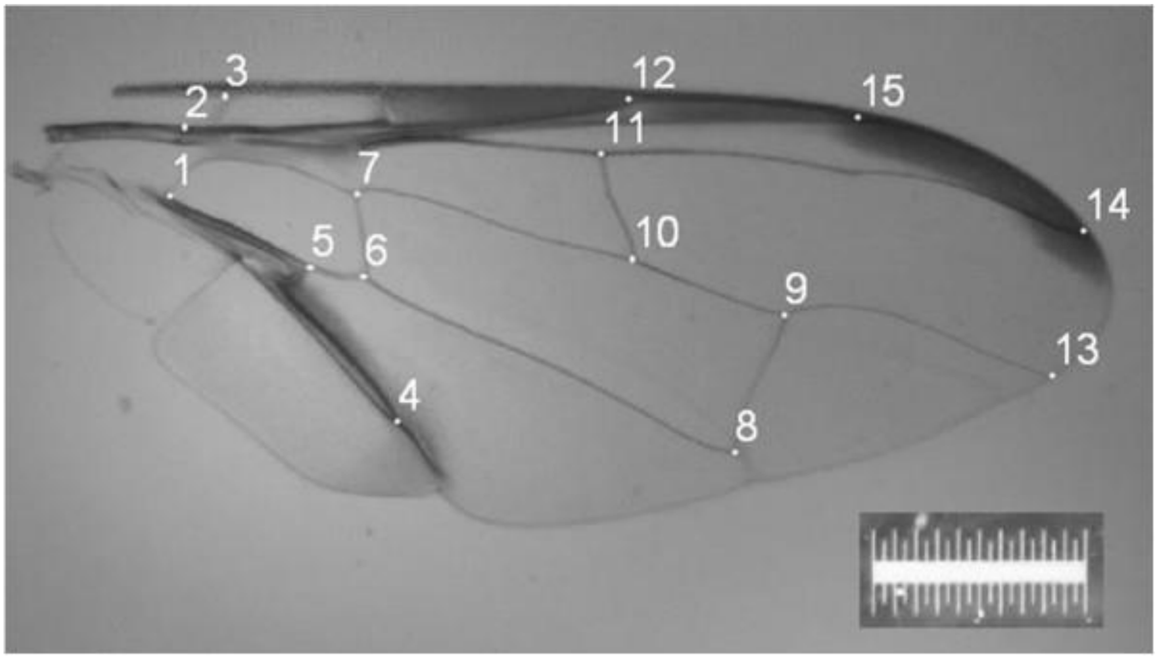

Supplementary Fig. S1

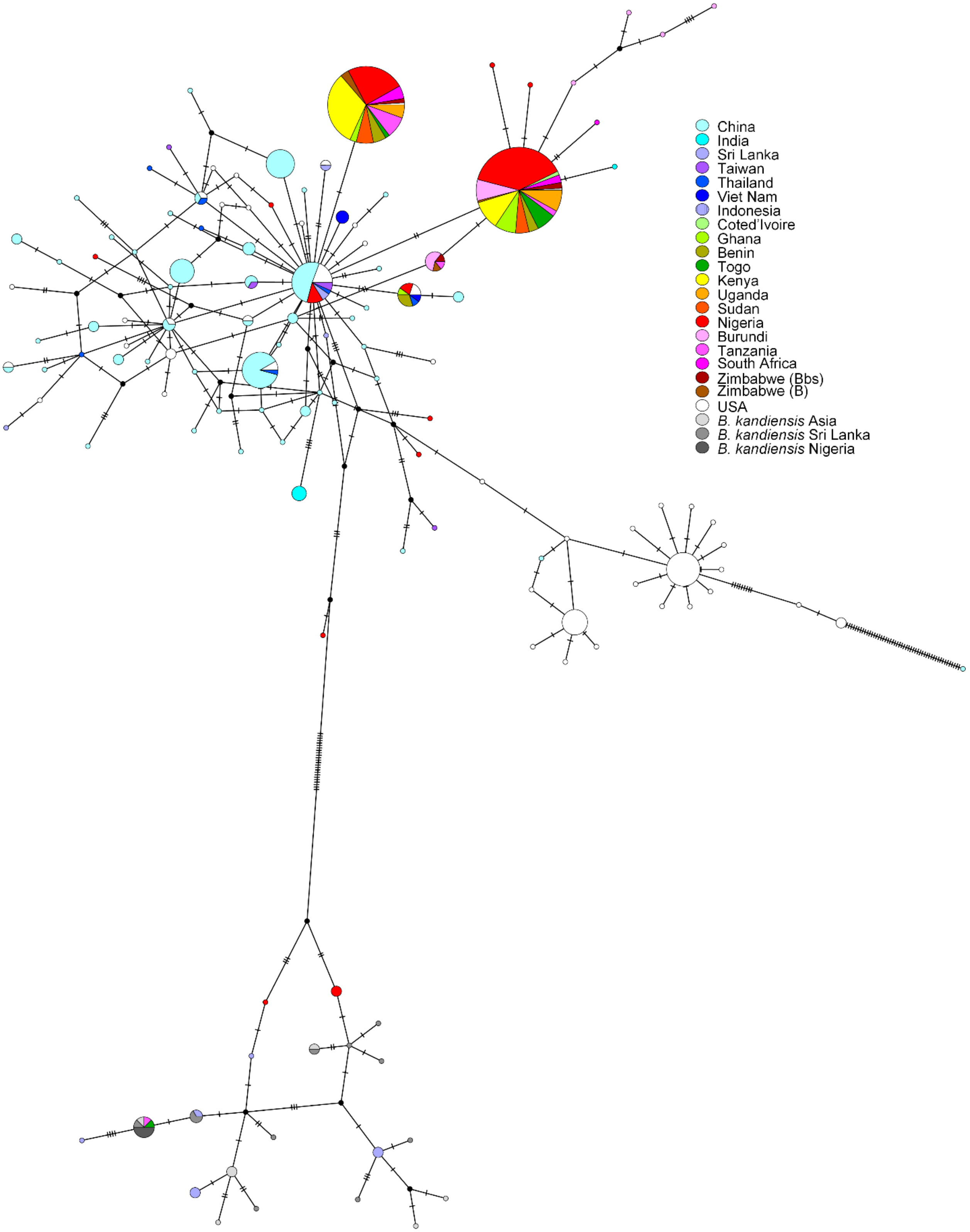

Supplementary Fig. S2

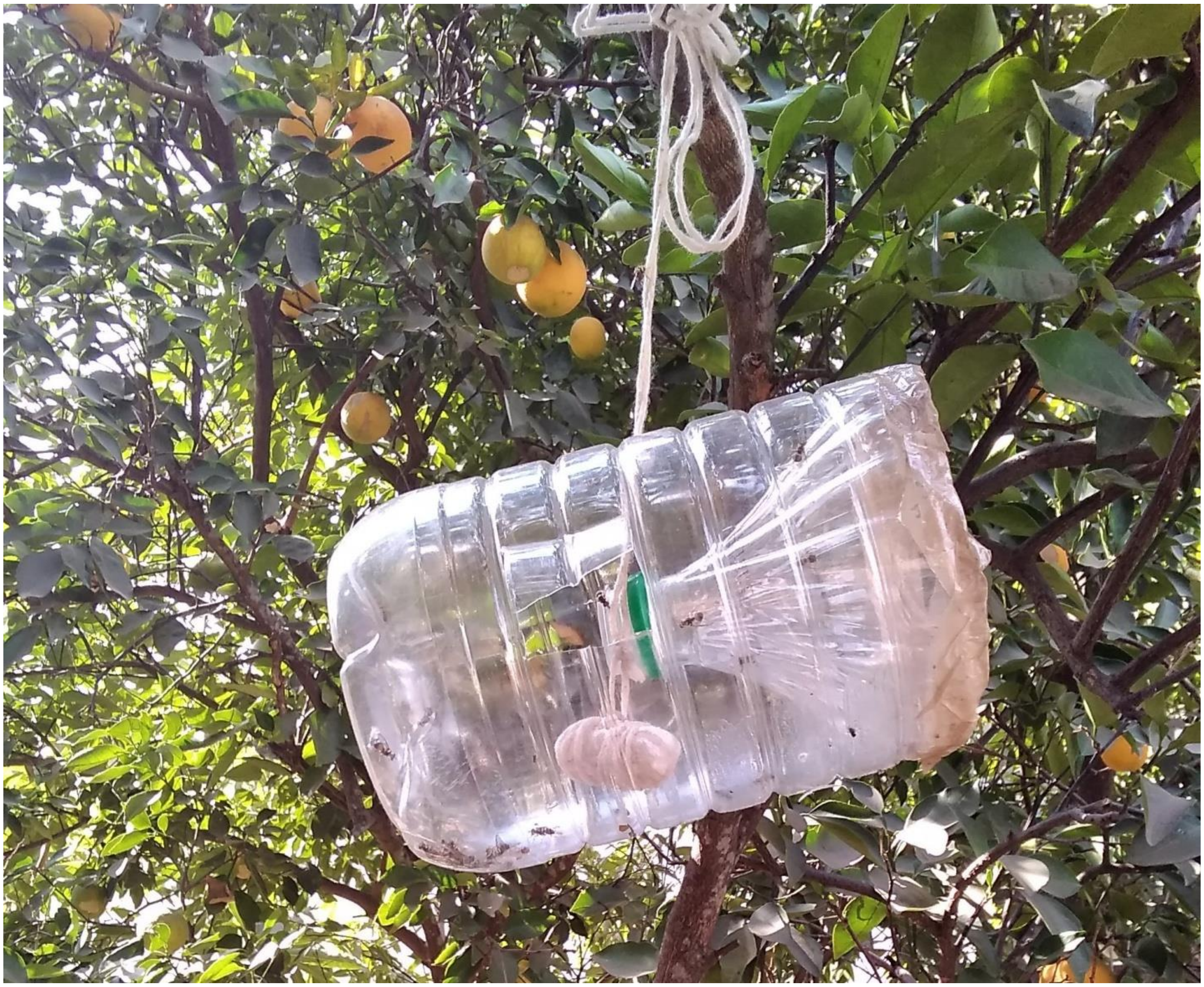

Supplementary Fig. S3
